# Aortic Sources of Embolism

**DOI:** 10.3389/fneur.2020.606663

**Published:** 2021-01-15

**Authors:** Elena Viedma-Guiard, Celine Guidoux, Pierre Amarenco, Elena Meseguer

**Affiliations:** Department of Neurology and Stroke Center, APHP Bichat Hospital, Paris, France Université de Paris, LVTS, Inserm U1148, Paris, France

**Keywords:** aortic arch, embolic source, stroke, stroke therapy, stroke type diagnosis, ESUS (embolic stroke of undetermined source)

## Abstract

Aortic arch atheroma is a frequent finding in ischemic stroke patients. Its role as a source of cerebral emboli or a marker of atherosclerosis is unclear. Transesophageal echography is considered the gold standard for its detection, whereas computed tomography angiography is a good alternative; magnetic resonance and positron emission tomography could be proposed to better analyze plaque vulnerability. Despite the interest in this condition, the optimal antithrombotic treatment remains uncertain, while intensive lipid-lowering therapy should be recommended. This review aims to offer guidance on patients with aortic arch atheroma, about its causal role in stroke, diagnosis, and treatment based on current available evidence.

## Aortic Sources of Embolism

Thrombi may form on ruptured atherosclerotic plaque in the aortic arch (AA) and are potentially responsible for artery-to-artery thromboembolism to the brain (1). Although this mechanism may cause ischemic stroke in some patients, AA atheroma (AAA) should generally be viewed as a marker of atherosclerotic stroke and cardiovascular disease (1). Another less frequent cause of aortic embolism is the “Cholesterol crystal embolization syndrome,” a systemic disease produced by embolization of multiple primary cholesterol crystals to the distal arteries of several organs (including the kidney) and the legs, causing ischemic and inflammatory damages (2).

In this review, we will mainly describe thromboembolism from AAA, its epidemiology, its importance in the etiological assessment of the stroke, and the therapeutic options.

The presence of AAA was described in post-mortem studies (3, 4). In an autopsy study performed in the general population in New Orleans, the prevalence of AAA increased with age: in patients aged 25–34 years, 4.9%; in those aged 35–44 years, 12.1%; in those between 45 and 54 years, 22.5%; and in those with 55–64 years, up to 33% (3).

The causality between AAA and stroke was first supported by an autopsy case–control study including 500 cases (4). They analyzed the presence of ulcerated plaques of the AA; in patients with cerebrovascular disease, prevalence was 26% compared to 5% in patients with other neurologic diseases (4). In those with brain infarction without an identifiable cause, ulcerated plaque prevalence was 61% compared to 22% in those with a potential cause (*p* < 0.0019) (4). Prevalence was also higher in older patients. Ulcerated plaques were found in 21% of patients with brain infarction aged between 60 and 69 years compared with 5% in controls (*p* = 0.019), 31% in those aged 70–79 years compared with 7% in controls (*p* = 0.01), 34% in those aged 80–89 years compared with 17% in controls (*p* = 0.128), and up to 50% in patients over 90 years compared with 33% in controls (*p* = 0.63) (4). The analyses also concerned the association of ulcerated AAA and extracranial internal carotid artery stenosis, and no relationship was found (4).

These data were supported by a multicenter autopsy study, where they found that 33% of the patients presented AAA (5). In this study, stroke was significantly correlated with complicated AAA [odds ratio (OR) 5.8, 95% confidence interval (CI) 1.1 to 31.7, *p* < 0.05], severe ipsilateral carotid artery disease (OR 3.1, 95% CI 3.1–45.3, *p* < 0.001), and atrial fibrillation (OR 3.5, 95% CI 1.1–9.9, *p* < 0.05), supporting the role of AAA in cerebral infarction (5).

The advent of transesophageal echocardiography (TEE) improved our understanding of this condition. TEE visualizes the proximal aortic segments, including part of the ascending AA, the horizontal part of the arch, and the proximal descending aorta. TEE studies described AAA in patients with transient ischemic attack (TIA) or stroke (6–8). The prevalence of AAA in stroke patients ranged 14–42% (1, 9, 10). In patients with TIA or minor stroke, AAA prevalence was also 40% (8). In case–control TEE studies, patients with severe AAA (plaque > 5 mm) had a higher incidence of stroke (36% in stroke patients vs. 4% in controls) (11) and peripheral embolism (27 vs. 9%) (12).

In 1994, a prospective case–control study including patients over 60 years found a strong and independent association between AAA and risk of ischemic stroke (13). Atheroma ≥ 4 mm was present in 14.4% of stroke patients compared to 2% of controls (patients having TEE for cardiac disorders). Statistical analyses were adjusted according to atherosclerotic risk factors; OR for ischemic stroke in case of aortic atheroma ≥ 4 mm was 9.1 (95% CI 3.3–25.5; *p* < 0.001) (13). Interestingly, 28.2% of patients with no obvious cause of stroke had plaques ≥ 4 mm, whereas in patients with other possible or likely causes of stroke, 8.1% had this kind of plaques (13).

Similar results were reported in another prospective case–control study, this one with community-based controls; data were analyzed according to vascular risk factors and high-grade carotid stenosis; results showed that atheroma in the ascending aorta and AA was a risk factor for cerebral ischemia; OR for simple atheroma was 2.3 (95% CI, 1.2–4.2) and while for complex atheroma OR was 7.1 (95% CI 2.7–18.4) (14).

The risk of recurrent stroke in patients with known AAA has also been evaluated (8, 15–20). A prospective study from the French Study of the Aortic Plaques in Stroke Group found an incidence of recurrent brain infarction of 11.9% and an incidence of all vascular events of 26 per 100 person-years in patients with an AAA ≥ 4 mm (15). According to AAA thickness, the risk of stroke recurrence increased: in patients with AAA 1–3.9 mm, it was 3.5 per 100 person-years, whereas in patients with AAA < 1 mm, it was 2.8 per 100 person-years (*p* < 0.001) (15). In this study, severe AAA was an independent predictor for recurrent brain infarction (relative risk, 3.8; 95% CI 1.8–7.8; *p* = 0.0012) (15). In a cohort study including 1231 patients with TIA or minor stroke, the incidence of recurrent vascular events at 1 year was 2.2% in patients without AAA, 4.1% in patients with moderate AAA (< 4 mm), and 6.6% in case of severe AAA (≥ 4 mm) (log-rank, *p* for trend = 0.003) (8). Other similar studies confirmed the higher risk of stroke recurrence in case of AAA (16–20).

One meta-analysis published in 2004 by Macleod estimates the OR of stroke in patients with severe aortic atheroma of 3.76 (95% CI 2.58–5.48), and results showed a remarkable degree of homogeneity between them (21). Another meta-analysis by Cui in 2014 had similar results; they found that AAA significantly increased the risk of stroke by almost four times (OR = 3.93, 95% CI 2.86–5.40) (22). All these data support the role of AAA as a potential cause of stroke. Moreover, the detection of microembolic signals using transcranial Doppler in stroke patients with AAA endorses the hypotheses of AAA as an embolic source (23–25). AAA is also considered to be a potential cause of embolism in patients with embolic stroke of undetermined source (ESUS) (26, 27).

However, AAA might only be a marker of generalized atheroma; its presence has been described in patients with other atherosclerotic disease such as coronary artery disease (28). Moreover, in a series of 1,200 patients with open heart surgery, 19.3% of the patients presented AAA ≥ 3 mm or with ulcerated or mobile components; the prevalence increased with age: from 9.6% in those aged 50–60 years to 32.6% in those over 80 (29). Furthermore, AAA is associated with vascular risk factors such as smoking, hypercholesterolemia, hypertension, diabetes, male sex, peripheral vascular disease, and elevated plasma levels of fibrinogen and homocysteine (1, 21, 30, 31).

The presence of AAA has also been described in patients with ischemic stroke and other potential causes of stroke such as intracranial atherosclerosis, small vessel disease, or atrial fibrillation. A previous study showed that the presence of simple AAA (< 4 mm or present in the descending aorta) was an independent predictor of intracranial atherosclerosis in patients with ischemic stroke (OR 1.94, 95% CI 1.17–3.21) (32). Interestingly, another clinical study showed that 36.4% of patients with AAA presented small vessel disease, including cerebral microbleeds, white matter intensities, high-grade perivascular spaces, and asymptomatic lacunar infarctions (33). Moreover, in a recent article in patients with stroke and atrial fibrillation, 38.4% had complex AAA (> 4 mm); the prevalence was higher in older patients and in those with diabetes and low high-density lipoprotein cholesterol (34).

The pattern of the infarction in patients with AAA has also been analyzed, magnetic resonance (MR) studies have shown that patients with complex AAA (> 4 mm or with a mobile component) had frequently small cortical lesions or subcortical single lesions (35). In another study in patients with cryptogenic stroke, the presence of multiple small lesions in multiple vascular territories were independently associated with vulnerable AAA, defined as > 6 mm, ulcerated plaque, or soft plaque (OR 33.18, 95% CI 4.26–258.45) (36).

The ASCOD classification analyzes the causality of AAA in stroke according to potential etiologic characteristics. In ASCOD, AAA is considered an atherothrombotic potential cause of stroke when a mobile thrombus is diagnosed and a possible cause if AAA is ≥ 4 mm without a mobile thrombus and is unlikely to be the cause of stroke if AAA is < 4 mm without a mobile thrombus (37).

In conclusion, it seems likely that AAA is a potential embolic site for the formation of the thrombus and responsible for ischemic stroke or TIA. It is also a marker of vascular risk, especially when AAA is over 4 mm.

## Detection of AAA

### Transthoracic Echography (TTE)

TTE can visualize the aortic root and proximal ascending aortic aorta. In selected patients, the AA and the descending aorta can be detected by suprasternal notch using TTE B-mode (38) and harmonic imaging (39). However, TTE is not very accurate in the detection of AAA.

### Transesophageal Echography (TEE)

**Figure 1:**
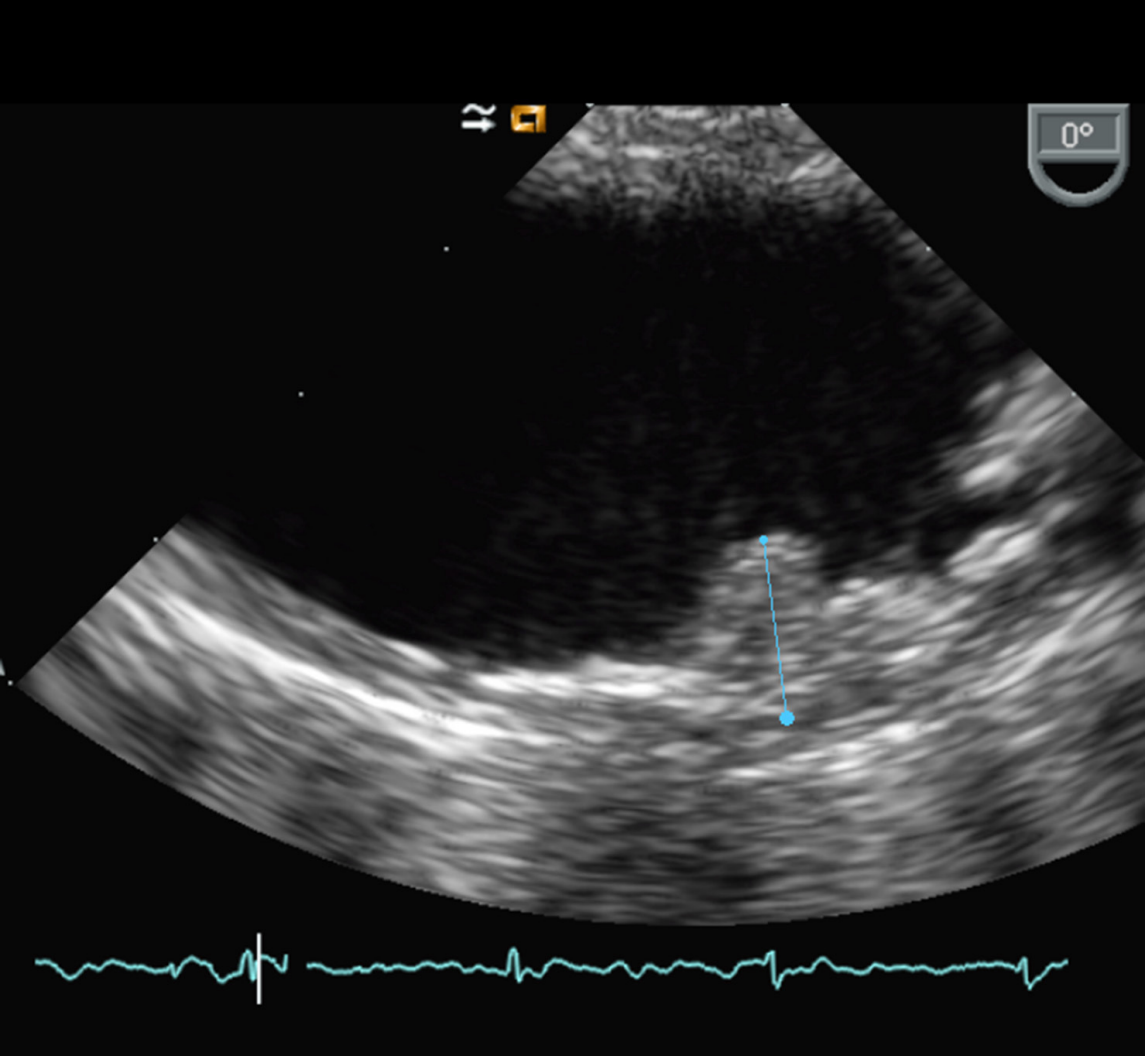
Transesophageal echography of the aortic arch showing protruding AAA.

TEE is the more common imaging technique used in the detection of AAA. This technique has a high sensibility and specificity (40). The quality of the images allows the detection of AAA, the measure of plaque thickness, the definition of its echogenicity, and the detection of ulceration, calcification, mobile components, or thrombus (Figure 1).

TEE is performed under local anesthesia of the oropharynx. The probe is introduced in the esophagus, after cardiac visualization; the probe should be rotated 120–180° counterclockwise to visualize the cross section of the entire thoracic descending aorta (40–45 cm from incisors). The arch is then imaged by slowly withdrawing the probe up to 18–20 cm from the incisors (41, 42).

TEE is usually a safe semi-invasive diagnostic technique. However, 0.1 to 13% of the patients describe minor oropharyngeal injuries, including hoarseness, sore throat, or odynophagia. Exceptionally major TEE complications including gastrointestinal bleeding or esophagus perforation may also occur (range from 0.2 to 0.5%). TEE-associated mortality has been estimated to be < 0.01%. Nearly 2% of patients under TEE have difficulties in the introduction of the probe, most of them because of a lack of patient cooperation and/or operator experience (98.5%); exceptionally, anatomical reasons can explain these difficulties (1.5%) (43).

### Computed Tomography Angiography (CTA)

**Figure 2:**
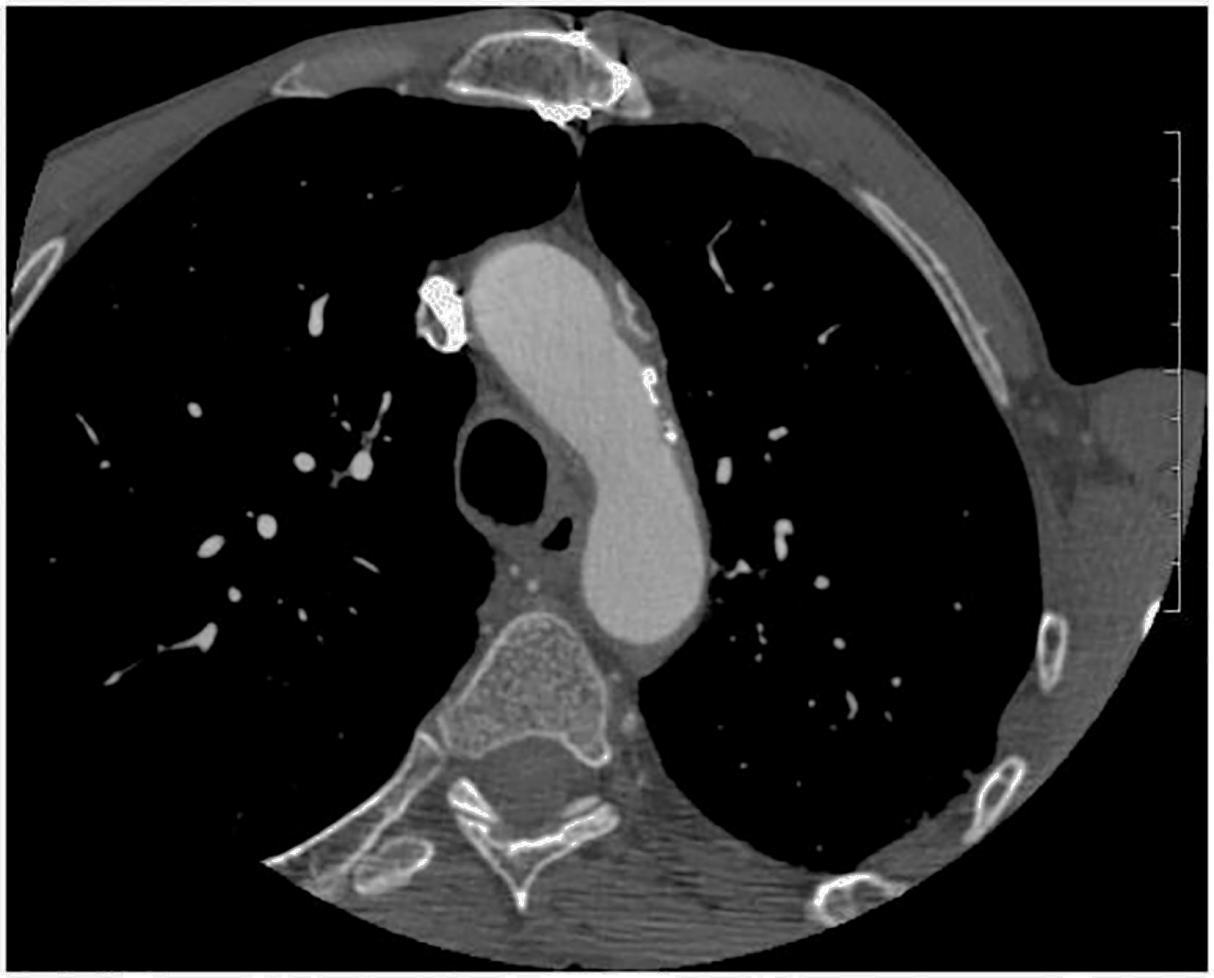
Computed tomography angiography showing an AAA.

CTA is used for non-invasive evaluation of the aorta and its major branches. The high-resolution helical CTA allows detection of protruding aortic plaques and identifies locations, plaque size, and plaque density. It is especially interesting in the study of areas not visualized by TEE (e.g., the distal ascending aorta) and is the best choice for detecting vascular calcification (Figure 2). On the other hand, it is not able to assess plaque mobility (44, 45).

Some studies have compared TEE and CTA for AAA detection. In one of them, dual-helical CT yielded a sensitivity of 87%, a specificity of 82%, and an overall accuracy of 84% (46). Other studies comparing both techniques found that CTA was not as sensitive as TEE (53%), but that it had high specificity (89%); however, in high grade AAA, specificity increased to 99%, but the sensitivity decreased to 23% (47).

Nevertheless, there are certain controversies as another study showed that CTA identifies more plaques throughout the AA and around the origins of the major cerebral arteries in particular, compared to TEE (48).

Depending on the patient's clinical conditions and available diagnostic possibilities, the physician will choose TEE or CTA for AA evaluation, taking into account that they provide sometimes complementary information.

### Positron Emission Tomography (PET)

Further information can be obtained from PET, which is used to localize hypermetabolism (fluorodeoxyglucose uptake by the plaque) identifying inflammation inside the atheroma plaque. This hypermetabolism is considered as a marker of instability, of higher risk of rupture and embolization. Novel PET tracers, which are designed to track active calcification, inflammation, hypoxia, or neoangiogenesis, may be potential markers for plaque rupture and cardiovascular risk (49).

### Magnetic Resonance (MR)

MR is useful for artery wall evaluation and identification of morphologic features of atherosclerotic plaques (calcifications, fibrocellular tissue, etc.), identifying markers of instability (size of the necrotic core or intraplaque hemorrhage) (44, 45).

Recently 3D-multi-contrast MR imaging has been used for the detection of the AAA in three dimensions, these images are high quality and they allow a better evaluation of the plaque, providing further information about the potential risk of future complications or even the effect of treatments and prevention measures during the follow-up. This technique is able to show in detail the plaque composition and can detect vulnerable AAA described as those with intraplaque hemorrhage or superimposed thrombi and those thicker than 4 mm (50, 51). MR has been compared with TEE and has been shown to either overestimate or underestimate plaque size, but, as CTA, is better than TEE to evaluate some characteristics such as penetrating atherosclerotic ulcers, intramural hematoma, or pseudoaneurysm formation (52).

## Treatment

### Antithrombotic Strategy

The optimal antithrombotic treatment in patients with AAA remains uncertain. In patients with ischemic stroke and AAA, several antithrombotic strategies have been tested to prevent recurrence. They included aspirin, combination of antiplatelets, warfarin, and combination of low-intensity warfarin with aspirin. Retrospective studies showed a potential benefit of warfarin over aspirin (17, 53). However, these data were not confirmed in match control studies or clinical trials (54–58).

There has been only one prospective randomized trial conducted specifically in patients with AAA and stroke. The Aortic Arch Related Cerebral Hazard Trial (ARCH) compared Aspirin plus Clopidogrel (A + C) vs. warfarin with target International Normalized Ratio (INR) 2.5 (range 2–3) in patients with ischemic stroke, TIA, or peripheral embolism, and AAA > 4 mm. Unfortunately, this trial was inconclusive because this event-driven trial did not meet its planned sample size and primary end point; it was prematurely stopped because of lack of recruitment. Another important pitfall was the lack of a third control group on aspirin only. The trial included 349 patients over more than 8 years; 7.6% of patients on A + C and 11.3% of patients on warfarin presented cardiovascular events, vascular death, or intracranial hemorrhage (log-rank, *p* = 0.2); however, this difference was not significant [adjusted hazard ratio (HR) 0.76; 95% CI 0.36–1.61; *p* = 0.5]. Major bleedings were similar with both treatments. Only vascular deaths were lower in the A+C arm compared to the warfarin arm (*p* = 0.013). There was no net benefit combining primary end point events and major hemorrhages between both groups (9.9 vs. 13.6% in the A + C and warfarin arms, respectively, log-rank *p* = 0.3; adjusted *p* = 0.5). However, in the group of patients with a time in therapeutic range > 77%, there was a suggestion that warfarin was superior to antiplatelet strategy, without reaching statistical significance (55).

The Warfarin–Aspirin Recurrent Stroke Study (WARSS) trial compared Aspirin vs. Warfarin in patients with non-cardioembolic stroke. A subgroup of patients from the WARSS with AAA showed a higher risk of stroke recurrence or death in case of atheroma and in case of bigger and complex plaques (26.7%), compared to patients with no plaques (10%) or small ones (16.5%) (56). They compared aspirin 325 mg vs. warfarin (INR 1.4–2.8), concluding that the risk was similar with both treatments (Aspirin 15.8% vs. Warfarin 16.4, *p* = 0.43), including patients with large plaques (HR 0.42, 95% CI 0.12–1.47) (56).

Rivaroxaban Versus Aspirin in Secondary Prevention of Stroke and Prevention of Systemic Embolism in Patients with Recent Embolic Stroke of Undetermined Source (NAVIGATE ESUS) trial compared Aspirin 100 mg vs. Rivaroxaban 15 mg in patients with recent ESUS. A subgroup of the NAVIGATE ESUS trial included patients who had TEE; they found that 29% of them presented AAA and 8% had complex ones (57). The annual risk of stroke recurrence was 7.2% in case of complex AAA, 4.2% in non-complexes, and 5.6% in case of non-AAA (57). They compared the use of Aspirin 100 mg vs. Rivaroxaban 15 mg. According to the treatment in each group, no difference was found in patients with no plaques, complex plaques, and non-complex plaques (57).

A recent meta-analysis including these three previous trials found no difference for treatment with anticoagulation vs. antiplatelet in AAA (OR 0.80, 95% CI, 0.40–1.62) (58).

In conclusion, antithrombotic treatment to prevent stroke or recurrence in patients with AAA remains uncertain, a single antiplatelet therapy strategy remains the gold standard, as generally recommended in guidelines for atherosclerotic cardiovascular disease. In case of mobile thrombus, short-term anticoagulation may be reasonable on a case-by-case basis.

### Hypercholesterolemia

The use of intensive lipid-lowering therapy is recommended after transient ischemic attack and ischemic stroke of atherosclerotic origin (59). In the Treat Stroke to Target Trial (TST), patients with previous stroke or TIA and atherosclerosis disease, including AAA, were treated by statins and/or ezetimibe to achieve LDL levels of 100 mg/dl vs. LDL-level < 70 mg/dl; levels lower than 70 mg/dl showed a reduction in cardiovascular events recurrence or cardiovascular death (59).

In a retrospective match-paired study during 12 years, they analyzed the effect in AAA and they found a reduction in clinical events; 12% of patients under statins compared to 29% of non-statin patients had recurrent embolic events (OR 0.3, 95% CI 0.2–0.6; *p* = 0.0004) (54). A small trial in patients with previous ischemic stroke has shown that 6 months of treatment by rosuvastatin diminishes the volume of high echoic plaques described in TEE (60). Clinical trials in patients with hypercholesterolemia and AAA have shown that long-term simvastatin (61), atorvastatin (62), and rosuvastatin (63) reduces vascular wall thickness in AAA measured using High-Resolution MRI. Simvastatin also attenuated plaque 18F-fluorodeoxyglucose PET uptakes and decreased the maximum standardized uptake values (SUVs) in patients with AAA, suggesting the role of statins in plaque inflammation (64). Furthermore, pitavastatin stabilized plaque inflammation evaluated by PET/CT in the thoracic aorta (65).

A recent study in animal models has shown that the association of alirocumab (antiPCSK-9 antibody), evinacumab (monoclonal antibody against angiopoietin-like protein 3), and atorvastatin reduced aortic plaques in the thoracic aorta and aortic root in mice (66).

Therefore, intensive lipid-lowering therapy should be recommended in patients with AAA.

### Surgical

Exceptional cases have been reported of surgical AA endarterectomy (67, 68). Moreover, in a study in patients under heart surgery and AAA, endarterectomy in AA was proposed; however, these patients did worst and presented a higher risk of stroke during the procedure (69). Therefore, there are no randomized evidence for recommending AAA endarterectomy or treatment of penetrating ulcers, and they should only be decided on a case-by-case basis.

## Conclusion

Severe AAA is an important risk factor for stroke, especially in those patients with AAA ≥ 4 mm, where the risk of stroke or peripheral embolism has an OR of 4. The prevalence of severe AA is about 30% in stroke, and it is increased with age. Moreover, recurrent stroke and cardiovascular disease are more common in patients with severe AAA. However, the role of AAA is still discussed as the cause of the stroke or a marker of atherosclerosis. We suggest AAA visualization in the workup of patients with ischemic stroke as it may alter the preventive treatment. TEE is the most accurate imaging tool, and CTA could be a good alternative when TEE is not available; MR and PET could be proposed to better analyze plaque vulnerability. Patients should receive high dose of statins. An antiplatelet treatment is mandatory, and sometimes dual antiplatelet therapy or anticoagulant may be considered.

## Author Contributions

EV-G and EM devised the project, the main conceptual ideas, proof outline, and wrote the manuscript. CG provided images. PA and CG provided critical feedback and helped shape the research, analysis, and manuscript. All authors contributed to the article and approved the submitted version.

## Conflict of Interest

The authors declare that the research was conducted in the absence of any commercial or financial relationships that could be construed as a potential conflict of interest.
